# Desistance From Intimate Partner Violence: A Conceptual Model and Framework for Practitioners for Managing the Process of Change

**DOI:** 10.1177/0886260514553634

**Published:** 2015-09

**Authors:** Kate Walker, Erica Bowen, Sarah Brown, Emma Sleath

**Affiliations:** 1 Coventry University, UK

**Keywords:** intimate partner violence, desistance, process of change, treatment and intervention, practical model

## Abstract

Intimate partner violence (IPV) is an international issue that social and criminal justice workers will encounter regularly. It has been identified that men can, and do stop using, or desist from, IPV although it is unclear how this process of change develops. This article introduces a conceptual model to outline how the process of desistance evolves and what it encompasses. Using thematic analysis of interview data from partner-violent men, survivors, and treatment facilitators, the resulting model demonstrates that the process of change is a dynamic one where men’s use of, and cessation from, violence needs to be understood within the context of each individual’s life. Three global themes were developed: (a) *lifestyle behaviors (violent)*: what is happening in the men’s lives when they use violence; (b) *catalysts for change*: the triggers and transitions required to initiate the process of change; and (c) *lifestyle behaviors (non-violent)*: what is different in the men’s lives when they have desisted from IPV. The purpose of this model is to offer a framework for service providers to assist them to manage the process of change in partner-violent men.

## Introduction

In order for social and criminal justice workers to effectively treat and manage perpetrators of intimate partner violence (IPV), we need to understand why men use violence in their relationships, how they stop using violence, and the mechanisms responsible for initiating this change. The aim of the current study is to examine and understand the factors that lead violent men to stop being violent, and the processes associated with this change, by examining the process of desistance, as outlined in the verbal accounts of IPV perpetrators, female survivors of violent relationships, and program facilitators. The aim is to develop a conceptual model identifying how the process of desistance is initiated, and the characteristics of the desistance process. The purpose is to provide a framework to help service providers set realistic treatment targets and facilitate the complex and difficult process of change.

Researchers have demonstrated that men can and do stop using violence in relationships; however, this research has broadly focused upon identifying whether and the extent to which this happens (e.g., Feld & Straus, 1989; Quigley & Leonard, 1996; Woffordt, Mihalic, & Menard, 1994). To date, no single theory or model has been developed that completely explains this process (for a review, see Walker, Bowen, & Brown, 2013). Göbbels, Ward, and Willis (2012) suggest that complete psychological and social accounts of the desistance process from when an individual makes the decision to stop using violence through to them becoming non-violent are missing from the literature. Such information is important and necessary to inform appropriate treatment and management plans.

The transtheoretical model of behavioral change (TTM), as a general model of change, has been applied to those who have used violence against an intimate. It has been suggested that perpetrators proceed through a set of stages that prepare them for, and assist them in, maintaining behavior change (Alexander & Morris, 2008; Eckhardt, Babcock, & Homack, 2004; Hellman, Johnson, & Dobson, 2010). Prochaska and DiClemente (1984) proposed via the TTM that as perpetrators reach different stages of the process, the levels of their desire and ability to change their behavior may differ. Indeed, Alexander and Morris (2008) found that in contrast to IPV perpetrators who were classified in the early stages of change (precontemplative) and who were less motivated and more resistant to change, those identified in the later stages of change reported greater improvement in levels of anxiety, depression, and anger control, when stage of change was assessed using the University of Rhode Island Change Assessment. However, previous research, in which the TTM has been applied to IPV, has adopted a quantitative approach (i.e., inferring the stage from scores) rather than focusing on understanding the triggers and mechanisms which underlie the process. It is, however, important to emphasize that the TTM is best represented as a spiral progression through the stages. That is, individuals rarely progress through stages of change in a linear fashion but rather relapse and revisit one or more stages (Prochaska, DiClemete, & Norcross, 1992). Likewise, it is not expected that desistance from IPV will follow a linear progression through identical stages for each individual, but that the process will be complex and involve a dynamic pathway on which there may be key identifiable phases that promote or inhibit the likelihood of desistance. Indeed, it is likely that while there may be certain elements experienced by all the men, the precise nature of these experiences is likely to be idiosyncratic. Sheehan, Thakor, and Stewart (2012) suggest that qualitative methodologies may be more suited to achieving a better understanding of such a complex and dynamic process, such as the process of change for perpetrators of IPV.

A small number of qualitative studies have examined components of the change process that IPV perpetrators have experienced during treatment or pre-treatment (e.g., Catlett, Toews, & Walilko, 2010; Curwood, DeGeer, Hymmen, & Lehmann, 2011; Pandya & Gingerich, 2002). However, none of the authors clearly report the length of time that individuals have desisted for, at the point of participation. Nonetheless, these studies do offer an insight into some of the processes that are likely to be relevant to those who successfully desist. Pandya and Gingerich (2002) in their microethnographic study (i.e., passively observing participants in treatment programs) examined the process of change during group-based therapy for six male IPV perpetrators (three completers and three dropouts). The completers identified that their experience of the consequence of their violent behavior was sufficient to drive them to change, whereas the non-completers felt that violence met their emotional needs. Furthermore, completers engaged with the program utilizing and practicing the tools and techniques offered, whereas non-completers did not. The researchers suggested that completers’ process of change involved engagement with the program, acknowledgment that they had a problem, and then transferring learning into practice. Curwood et al. (2011) examined interview data from a larger sample of men (*n* = 42) prior to them attending group treatment. The authors sought to understand the processes of men’s behavior change following arrest, being charged, and then subsequently convicted of domestic violence (for the first time). The study utilized grounded theory to explore the narratives of men, using a process of open inductive coding (i.e., no preconceived coding framework was utilized) to construct a theoretical understanding of the men and their worlds, based on the men’s own lived knowledge and experience. Change was found to be a complex procedure that began well before treatment commenced, and the process of change had to be negotiated at several ecological levels: the individual (e.g., stress and anger management), the interpersonal and the relational (e.g., improved communication and patience), and the external (e.g., employment status or career aspirations). The findings from these two studies offer some important insights into change processes. However, their contribution is limited as it is not known whether any of the men in the samples were still using violence, so findings need to be taken with caution as they may not relate to desistance at all.

Only two studies have examined men’s experiences of how they stopped using violence against their intimates, where a measure of cessation of violence was included. Gondolf and Hanneken (1987) interviewed 12 men attending a treatment program, who were classified as reformed batterers on the basis of being non-violent for 10 months, to establish a phenomenological view of how men stop their abuse. The actual methodology undertaken to achieve this aim was not stated. However, the authors reported that the process of change included the acceptance of responsibility, becoming empathetic, and the redefinition of perpetrators’ “manhoods,” (e.g., that masculinity is associated with being able to show emotions and not the stereotypical macho male image which the men previously related to their use of violence). Scott and Wolfe (2000) purposefully sampled nine men deemed by themselves, facilitators, and partners to be successfully changing on the criterion of being violence free for six months. All men had recently completed treatment and were interviewed using semi-structured interviews designed to elicit personal stories of how they changed their abusive behaviors. Coding began with 28 a priori coding categories based on nine theories applicable to understanding change in abusive behavior: feminist, social-cognitive, personality, systems, attachment, deterrence, the health-belief model, the theory of reasoned action, and the information-motivation-behavior skills model. For example, one of the a priori coding categories was “communication/assertiveness” which was implicated as important by social learning theories and was applied when reference was made by the men to learning communication skills (listening, body language, awareness) and learning assertiveness skills or using an assertive response as an example. Four variables, taking responsibility, developing empathy, reducing dependency, and improving communication skills were endorsed by 75% of the men interviewed, suggesting these factors are particularly important in behavior change. A key limitation of these studies is that the findings are based on relatively small samples of men. In addition, without a comparison sample, it is not possible to draw conclusions as to whether the components of change identified are directly due to cessation of IPV or a result of completion of this specific program.

It is therefore the case that the existing literature that has examined how men desist from IPV is limited not least in its size but also in the extent to which a thorough and complete understanding of the process of change in IPV has been achieved. Research from parallel fields may contribute further to this understanding. For example, Göbbels et al. (2012) developed an integrated theory of desistance from sex offending (ITDSO). Some aspects of this theory may be relevant to IPV. ITDSO describes the desistance process in four phases: (a) decisive momentum (initial desistance), (b) rehabilitation (promoting desistance), (c) re-entry (maintaining desistance), and (d) normalcy (successful desistance over a long period of time). This suggests that desistance is a dynamic process where internal and external variables, and environmental, social, and psychological processes interact. A particular strength of this model is the identification that it is necessary to include a temporal dimension when developing theory, because desistance is a process rather than a discrete point in time. In addition, the authors emphasize the role of agency in the process. Although the ITDSO is in its infancy and remains work in progress, the theory may be useful for stimulating theory building in relation to desistance from IPV.

### Research Aims

Based on the literature reviewed, the aim of the present study was to further our understanding of the process of change that male IPV perpetrators experience to achieve desistance. In so doing, a conceptual model of the desistance process was derived from the qualitative analysis of accounts from multiple perspectives (desisters, persisters, survivors, and facilitators).

## Method

### Design

For the development of the conceptual model, a qualitative approach was selected. This approach is well suited to exploratory research studies, particularly where the focus is on achieving an insight into people’s attitudes, behaviors, and motivations (Ritchie & Lewis, 2003). Semi-structured interviews were undertaken, using specific questions developed by the research team to keep uniformity across all participants. Questions were constructed to get an insight into stability, frequency, severity, and type of violence that the men have used as it has been suggested that these factors may be related to desistance and persistence (e.g., Caetano, Field, Ramisetty-Mikler, & McGrath, 2005; Quigley & Leonard, 1996). In addition, as desistance is generally seen as a process and not a static point in time (Maruna, 2001), the questions focused on getting the participants (perpetrators and survivors) to give a timeline of events that encapsulated what their life was like before they had used violence, when they were using violence, and during periods when they were not using violence. Likewise, the facilitators were asked questions about what the perpetrators were like when they first came into contact with them through to the current time or when they finish their contact with them. As researchers have suggested that desistance is a complex process that is likely to involve an interplay between individual characteristics and social factors (Healy, 2010) where both structure and agency have a role (Farrall, Sharpe, Hunter, & Calverley, 2011), questions focused on asking about individual characteristics and subjective factors as well as contextual factors associated with persistence and desistance. All interviews followed the same structure and questioning format (interview schedules available on request from authors) although questions were worded differently depending on whether the participant was a desister, persister, survivor, or facilitator. However, to elicit detail, prompts were used where appropriate, such as asking individuals to explain, expand, give more detail, and give examples or by rephrasing the question to elicit a more detailed response. The data generated were analyzed using thematic analysis (TA). The themes were used to develop a conceptual model that represented the process of desistance from IPV and a framework for treatment targets and managing the process of change.

### Participants

Men who identified that they had used violence in their relationships were classified, based on their use of physical violence in their lifetime and the past year (measured on The Revised Conflict Tactic Scale; Straus, Hamby, Boney-McCoy, & Sugarman, 1996), as either desisters (used physical violence in their lifetime but not in the past year) or persisters (still using physical violence in their relationship). Feld and Straus (1989) have highlighted that if an individual remains violence-free for 12 months, this is clinically significant. All desisters met the requirement of being violence-free for at least 12 months based on their self-reports. The survivors were all females who had experienced physical violence from their male partners. Three of the survivors had left the relationships due to the violence and therefore their partners had persisted with physical violence throughout that relationship. For the remaining four survivors, their partners had been violence free for at least a year, based on the men’s use of physical violence.

In total, 13 male desisters (*M*_age_ = 38.0 years, *SD* = 10.3), nine male persisters (*M*_age_ = 36.0 years, *SD* = 10.3), nine (five female and four male) Offender Managers/Program Tutors (to be referred to collectively as facilitators; *M*_age_ = 43.7 years, *SD* = 9.1), and seven female survivors (*M*_age_ = 49.14 years, *SD* = 7.19) were interviewed. Participants were recruited from rehabilitation programs in England that the men were attending, or were waiting to attend, or had completed. The men were court-mandated through probation to attend treatment (*n* = 10) or they had self-referred to a community program (*n* = 12). The facilitators were also recruited through these organizations, as were survivors who were identified through women support workers.

No names are used when presenting the results, to maintain confidentiality for those who were interviewed. However, to identify which group each individual comes from, when presenting quotes from the data, the following coding is used: S for survivor, D for desister, P for persister, and F for facilitator.

### Data Collection

Ethical approval was obtained from Coventry University’s Research Ethics Committee and the National Offender Management Service (NOMS) through the Integrated Research Approval System (IRAS). All participants were interviewed on a one-to-one basis in a private room. For the offenders and facilitators, this was completed at the location where they attended or delivered treatment programs, the survivors were interviewed in their own homes. The interviews were all semi-structured but followed slightly different formats dependent on the type of interviewee. The basic organisation of the interviews for the IPV men included background information and details of the use of violence within relationships and the process of change. These questions were tailored depending on whether the man was a desister or persister. The survivors and facilitators were asked about their backgrounds regarding either working with offenders, or their experiences as victims of IPV and their views on the process of change in perpetrators.

### Data Analysis

TA (Attride-Stirling, 2001, Braun & Clarke, 2006) was used to analyze the data. TA is a flexible approach that can be applied across a range of theoretical and epistemological approaches (Braun & Clarke, 2006) and allows the researcher to develop independent themes and associated subthemes. For the purpose of the current research, analysis involved understanding both the role of individuals in constituting the social world in relation to IPV and the independent mechanisms that may also form part of the process, meaning that critical realism was the epistemological position taken.

All of the interviews were transcribed manually and verbatim. The TA of the text followed well-known techniques in qualitative analysis and developing thematic networks that summarize the main themes found in the data set. These themes were analyzed through organization and description as well as by interpretation of the various aspects of the research topic under investigation (Boyatzis, 1998). TA enabled the researcher to uncover salient themes within the text at numerous levels and the thematic networks facilitated how to structure and depict these themes (Attride-Stirling, 2001). Networks are built from three classes of themes: (a) basic themes that are the lowest order of theme derived for the textual data, (b) organizing themes that are middle-order themes and are represented by basic themes, and (c) global themes which are superordinate themes that encapsulate the principal concept in the data as a whole. To support the themes that were generated, a balanced presentation of the experiences of those interviewees was provided to promote trustworthiness of the data (Silverman, 2000) and direct quotes are provided to promote verifiability (Murphy & Meyer, 1994). In addition, to promote reliability and validity, a second independent researcher cross-checked coding strategies and interpretation of data.

## Results and Discussion

### Conceptual Model

The resulting conceptual model confirmed that desistance from IPV is a dynamic process that gradually unfolds over time (e.g., Maruna & Roy, 2007) and not simply a static spontaneous unique event. As this is a process (with potential false starts for some), the model needs to encapsulate the circumstances surrounding men’s use of violence, the factors that initiate the process of change, and how men’s lives are different when they have stopped using violence. The conceptual model comprises three main elements:

1. Lifestyle behaviors (violent): “Old way of being”
2. Catalysts for change
3. Lifestyle behaviors (non-violent): “New way of being”

**Figure 1.**
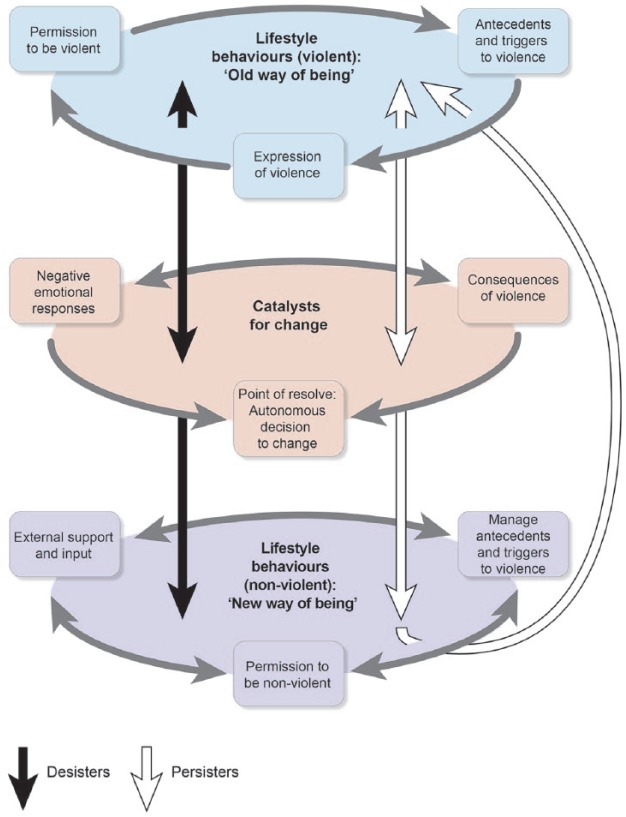
Conceptual model.

The conceptual framework, with these three global themes and their composite organizing themes, are presented diagrammatically in Figure 1. The framework demonstrates that the transition of persistence to desistence is not a linear process, but instead reveals two different lifestyle phases representing persistence ("old way of being") and desistance ("new way of being"). Progressing from persistence to desistance involves experiencing “Catalysts for change” in the form of a range of triggers that initiate the move to desistance. As can be seen, the elements of this model are all intrinsically linked.

The model embeds the use of violence or desistance from violence within a person’s life context. The framework deliberately captures the life context on the basis that the processes of persistence or desistance cannot be adequately explained if they are separated from the life context and the individual’s “lifestyle of behaviors,” within which these processes occur. Furthermore, each component (e.g., triggers) of the model is rooted within the individual’s life context. The framework describes how these men have different “ways of being” depending on whether they are persisting or desisting from their use of violence. Practitioners therefore need to understand what each individual’s way of being is, to treat and manage them effectively.

The remainder of this article will provide an overview of the global and organizing themes shown in Figure 1. The organizing themes developed comprise numerous subthemes, but it is beyond the scope of this article to present these individually. (Please contact the lead author for more information if required.)

### Lifestyle Behaviors (Violent): “Old Way of Being”

The first element of the framework reveals the lifestyle behaviors of persisters: how persisters view their worlds, how antecedents to violence develop, and how they respond to latent and/or acute triggers that exist in their worlds on a day-to-day basis. It is how the men view and respond to these factors that represents their ways of being, which includes the use of violence, that is, the use of violence among persisters is not conceptualized as isolated incidents, but manifestations of each individual’s “way of being.” This “way of being” is the first global theme that is represented by the three organizing themes detailed below.

#### Antecedents and triggers to violence

This theme is made up of situations, contexts, and risk factors that lead to a violent reaction, and include antecedents that are initially temporally distal but then over time become proximal factors, that is, they build up over time and accumulate to result in a violent act. Violence seems to occur in response to numerous factors (e.g., control, alcohol, life stressors). Some factors are latent, but accelerate and grow until they reach a tipping point, whereas others factors are acute, that is, instantaneous triggers. An example of an acute trigger was a lack of self-control, a risk factor of IPV commonly identified in the literature (Kerley, Xu, & Sirisunyaluck, 2008; Payne, Higgins, & Blackwell, 2010). For example,

**D:** And the way she looked at me. It just sparked me into violence . . . it was just, it was a sense of you know, hmm I don’t know it was a loss of control, it was lost control.

There is considerable evidence that alcohol has a variety of functional roles in relation to the use of violence, such as, impairing cognitive functioning, limiting the capacity to comprehend social cues, and increasing the risk of violence for those with aggressive predispositions or deficient social perceptual processes (Clements & Schumacher, 2010; McMurran & Gilchrist, 2008). The dangers associated with alcohol in the context of a violent relationship were also widespread within the current data, for example,

**S:** Drink was a, he would drink a lot yeah, if he was drunk then I had to be careful.

Examples of other latent triggers were life stressors. Children, employment, and financial strains are stressors often associated with the use of violence in a relationship (Cano & Vivian, 2003; DeMaris, Benson, Fox, Hill, & Van Wyk, 2003; Kyriacou et al., 1999), and were identified in the current study. In the example below, it was financial problems.

**P:** Hmm it was always about money. The only subject I remember arguing about with her in that time . . . I’d feel myself getting tense and wanted to hit something.

These themes represent the issues present in the men’s lives, which for them, at that point in time, resulted in violence. These latent and acute factors need to be the target of intervention through elimination and management and by teaching the men different reactions to certain contextual and situational triggers. Therefore, intervention needs to concentrate on teaching the men not to reach the threshold point, that when crossed, results in them using violence.

#### Expression of violence

This theme highlighted the range of violent behaviors enacted by the men. These included physical violence such as pushing, shoving, hair pulling, and incidents where men slapped, hit, punched and “beat up” their partners. The men reported using threatening and abusive (e.g., physical intimidation) behaviors. The use of emotional and verbal violence was very common and found across all interview data.

#### Permission to be violent

This organizing theme represents male perpetrators justifications that enable them or give them permission to continue using violence. The justification (or permission) is strong enough to prevent them from moving on to the desistance pathway, and is a barrier to change. The men create justifications in several ways. One way was to convince themselves that their behaviors were *not* violent or abusive. In doing so, this reframed their behaviors as not wrong or out of the norm. This meant that the behaviors were not an issue that needed to be thought about further, challenged, or changed. This was present in all of the accounts and is supported by this comment from a survivor.

**S:** He didn’t believe that he had done wrong, he doesn’t believe he ever raped me. In his world it was a sexual encounter between a husband and wife.

Other men blamed their partners saying that violence occurred due to provocation or in self-defense. The men contextualized their violence to normalize it either by explaining their violence was “just” arguments normal within *their* relationships:

**D:** It was normality in our normal relationship that a bit of push and shove; or normal in *all* relationships in general.

**D:** That was just like normal life. I mean my Dad was violent to me Mum, me brothers and sister in law were violent to each other.

The men also gave themselves *permission to be violent* by assigning certain traits and characteristics to themselves that enable them to take on the identity of “IPV perpetrator.” Such characteristics included psychological issues (**P**: I am bordering on being a little psychotic), having an aggressive nature **(F:** [*The offender*] classed himself as “I am an angry man and that’s how I am”), and having trust and jealousy issues (**D:** I just didn’t trust nobody. I didn’t trust, I didn’t trust ****[*partner*] and I didn’t trust me mates). What is important in this theme is how the men use contexts, justifications, and their identities to avoid or remove psychological discomfort (or cognitive dissonance; Festinger, 1962). This enables them to achieve psychologically comfortable states (consonance) and gives them permission to continue to use violence. This needs to be considered in relation to treatment because this psychological position inhibits their ability to understand that there is a need to change.

### Catalysts for Change

Desisters and persisters all experienced the first part of the model: Lifestyle behaviors (violent) and their accounts reveal the factors and issues that create and maintain this “old way of being”. The most important part of the process of desistance is the bridge between this Lifestyle behaviors (violent) and the latter Lifestyle behaviors (non-violent), as it comprises the triggers and transitions that the men experience, and which directly or indirectly activate change. These internal and external factors function as stimuli or catalysts for change. Rather than a single, defining moment or incident that enables the men to spontaneously desist from IPV, the triggers accumulate and gain momentum over the course of time. When the triggers are perceived as important enough, they lead the men onto a new pathway of Lifestyle behaviors (non-violent). This global theme therefore represents the part of the conceptual framework that details the factors associated with initiation of the desistance process.

#### Consequences of violence

Some triggers were external and included events, situations, or incidents that occurred and progressively activated the men’s thought processes toward recognizing the need to change. Examples are the impact of violence on their family (children witnessing violence, seeing damage done to partner) or criminal justice involvement either in the form of fear of prison or actual arrest. All of the desisters experienced an accumulation of different triggers to desistance. The precise form and quantity of triggers differed from person to person, but they gained momentum over time and instigated thoughts that change was required. One of the men clearly identified this accumulation.

**D:** There wasn’t one big shocking event that you suddenly thought, right I need to change. It was kind of a process of the police were involved, probation were involved, girl friend saying you should be something about it, IDAP come along.

#### Negative emotional response

This trigger included the negative emotions of guilt, shame, and fear which occurred as a consequence of the use of violence. These negative emotional responses cannot be considered in isolation. They increased in intensity over time until their presence reached a threshold at which point they began to act as a form of psychological punishment.

**D:** After that I felt disgusted with myself. Through actually hitting someone let alone a woman, you know made me feel ill really. And that’s something that I’ve got to deal with for the rest of my life.

#### Point of resolve: Autonomous decision to change

Desistance was only initiated following an interaction between the external structural factors (consequences of using violence) and agency (internal negative emotional responses). However, each man reported experiencing an intrinsic trigger that came from within and which stimulated the recognition that change was required. This was not a spontaneous event, but resulted from experiencing several triggers. The men reached a point of resolve and made an autonomous decision to change. Without this happening, they could not start the process of desistance. As one of the men commented:

**D:** I was on the verge of insanity, I was angry all the time . . . I just thought I can’t go on like this anymore . . . I needed to get it sorted . . . It (*decision to change)* was all from within me.

Change is triggered by an accumulation of external triggers over time which become internalized and facilitate the development of intrinsic autonomous motivation. Autonomous motivation originates from the self and fulfills personally relevant goals (Deci & Ryan, 2012) and has been found to be positively associated with various types of behavior change, such as health-related behaviors, and exercise and dietary behaviors (Ng et al., 2012; Silva et al., 2011; Teixeira, Patrick, & Mata, 2011). This has not been explored in relation to IPV but the current data illustrate that this form of motivation was a key trigger for the men; they made conscious (or autonomous) choices to change. This should be explored by practitioners further in relation to IPV, as it may be a crucial point in the process and therefore needs careful consideration in relation to intervention.

### Lifestyle Behaviors (Non-Violent): “New Way of Being”

This new stage in the desistance framework is where the men actively participate to enable them to desist from violence. This cannot be achieved passively. This new way of being for the men is where a lifestyle that includes violence is replaced by one that is non-violent. However, the different elements identified in this lifestyle are not experienced in a specific order and are elements that run concurrently. Within the model, “Lifestyle behaviors (non-violent)” is a state that the desisters have experienced for at least a year and represents what is happening within their “life” context now that violence is not present. This demonstrates a different view of the world: how triggers to violence are managed and how being non-violent is now conceptualized as the manifestations of the individuals’ new ways of being. It is more accurate to suggest that the conceptual model at this point depicts short-term change, as the new behaviors may not be completely entrenched. Indeed, this remains a process of active behavior management. Some persisters may have experienced some elements of this pathway, but were unable to sustain this way of being over time and so returned to their old ways of being, in using violence. For some, they have not managed to move off the violent pathway, in part because they have not experienced as many external triggers. In addition, such triggers have not been perceived by these individuals with the same level of importance as others, so that an internal trigger has not been activated that has stimulated and initiated the process of change.

#### Managing antecedents and triggers to violence

The first part of achieving desistance is managing the antecedents and triggers to violence. The men recognize (remove denial, minimization, and blame) and pre-empt the triggers (actively maintaining consonance and avoiding dissonance), so they can handle situations differently and not resort to using violence. Other factors included managing day-to-day stressors and becoming proactive in the creation of stability in their lives. For example:

**D:** One of the first things I did to change the situation was to get rid of everything that was a factor causing the problem. So I started off with the smallest the first, the housing situation . . . And then I sorted out the debt situation . . . My job, my job was another factor . . . I went through all the factors and knocked them off one by one.

Managing the antecedents and triggers to violence involves using several different strategies: for example, better communication techniques, reducing alcohol, pre-empting triggers to violence. This means the men look for ways to change the situations and respond differently to what had been the norm, that is, responding with violence. A focus therefore needs to be placed when working in IPV to teach individuals to mange situations differently to achieve a different outcome, that is, one that is not violent. The can be achieved by helping men understand the context of their risk from situational to contextual factors, and by providing assistance to help them address the issues most functionally linked to their use of violence.

#### Permission to be non-violent

This is similar to the corresponding theme found in the lifestyle behaviors when the way of being was violent (*Permission to be violent*), and it serves a similar purpose. However, on this occasion, the permission is such that it encourages and justifies why the men are non-violent in their relationships, as opposed to justifying and maintaining IPV. The individuals have had to make radical changes in their underlying beliefs or theorizing about their behaviors. A prerequisite of this paradigm shift is *awareness* that there is a problem or issue that needs to be changed. In this part of the process, one conceptual view of the world needs to be replaced with another. The main ways that the men do is by first seeing their behavior as abusive:

**D:** I absolutely, well admit and recognize, well no, recognize then admitted. It’s acknowledgment . . . I think the moment of recognition is crucial.

However, they also need to look internally and become accountable for their own behaviors and actions (which they have the abilities to change). This again involves a paradigm shift, as a realization is required that the focus needs to be completely on the self:

**D:** For my behavior, I have to be entirely responsible for my behavior and my responses were inappropriate.

As can be seen, the men look to the self (i.e., internally) and then attribute to themselves characteristics, behaviors, and beliefs that are aligned with individuals who are non-violent. Part of the process relates to the active management of propensity as the men adorn themselves with characteristics incompatible with those of IPV perpetrators. In their old ways of being, negative-internal attributions (characteristics and behaviors) were associated with persistence in offending, that is, the men were unable to desist as negative events were associated with their internal characteristics—“this is the way I am” (*Identify self as agent of abuse*). The men therefore construct this explanation for their use of violence and such explanations have been associated with continuing an action over time (Braithwaite & Braithwaite, 2001). However, in the new ways of being the men give themselves permission to be non-violent by ascribing *not* being violent to internal attributes and they execute processes of positive-internal attributions. For example:

**D:** It’s about your own choices and your own behavior. Now I’m calm, placid, normal . . . They would describe me as a growler, I would get angry and growl at them . . . now I don’t growl no more I’m very calm.

#### External support and input

This final organizing theme acknowledges the need and requirement of external support networks as an integral part of the desistance process. While the men have to look internally to acknowledge and change their behaviors, this needs to be supported by partners, families, and treatment providers. External support seems to offer both guidance on what needs to change and how this can be achieved. External support also appears to be crucial in helping the men to sustain their new ways of being, which enables the men to continue on the lifestyle behaviors (non-violent) and not return to the lifestyle behaviors (violent).

Treatment was acknowledged throughout as being an important element needed to assist the men on their non-violent pathways. This finding has been previously identified in IPV men (e.g., Daniels & Murphy, 1997; Gondolf & Hanneken, 1987; Silvergleid & Mankowski, 2006) as treatment is particularly influential at encouraging the men to recognize what is abuse and encouraging them to move away from denial and blame toward responsibility and action. One man recalled:

**D:** My whole outlook on relationships changed, the whole outlook . . . going to this group at the ***** has changed me big time it really has, just the whole outlook on life really.

The group was also found to be a particularly strong support system. This finding has also been reported by other researchers (Sheehan et al., 2012; Silvergleid & Mankowski, 2006) who have found that relationships with other men in treatment groups facilitates behavioral change through positive feedback that reinforced and shaped behavior change, and through manifesting the feelings in the men that they were not alone in this. The men also needed support and encouragement from others to change and this included family, friends, or their partners:

**D:** And the more and more I got praised . . . and more and more hearing good things from **** (*partner*) . . . it meant enough to me to sort give me that motivation to keep going.

This final theme gives an insight into the external support networks that were important mechanisms during the process of desistance. This factor runs alongside the two themes, and all three make up “Lifestyle behaviors (non-violent).” Once at this stage of the process, the men have recognized their behaviors are abusive and taken responsibility for their violence. They look to identify themselves as agents of change, put different strategies in place, and adopt mind-sets to stop using IPV. For practitioners, it is fundamental to understand how external support is required to assist this process and help with the maintenance of violence-free lives.

### Implications for Practitioners

This model explains how violence fits within the context of perpetrators’ lives, and examines what is different when violence is removed. It deliberately embeds desistance from violence (and persistence) within the person’s life context as the desistance process cannot be adequately understood in isolation of this. This therefore offers practitioners a practical tool to understand what it is about individuals’ lifestyles, behaviors, and attitudes that means violence is a feature of their relationships, and more importantly how this changes when men’s relationships are violence free.

This model highlights that the path from persistence to desistance is not a straightforward linear journey that is shared by all IPV offenders, but is complex, dynamic, and idiosyncratic. The process of desistance is distinct for each individual, and this requires individual assessments for each IPV perpetrator. Such assessments must identify the contextual and situational factors associated with each individual’s use of violence, and also the functional relationship between antecedents and violent behaviors (i.e., what is his current lifestyle behaviors, and their autonomous reason for change). This will allow practitioners to tailor treatment based on individuals’ situations, motivations, and therefore risk. Such an approach would be consistent with the Risk-Need-Responsivity (RNR) model of rehabilitation, from which numerous effective treatment programs have been developed for a range of crimes including non-intimate violence (Andrews & Bonta, 2003; Andrews, Bonta, & Wormith, 2006; Andrews & Dowden, 2006). Based on the model presented, treatment needs to match offender’s individual risk level. Risk can be established based on current lifestyle behaviors being experienced. As risk increases (e.g., in the stage where individuals give themselves permission to be violent, not recognizing their behavior as violent, stressors in relationship), the extent of treatment required to promote desistance will increase. Individuals at different stages of the process of desistance need different types of treatment, for example, assisting with maintenance of “permission to be non-violent.” Therefore, treatment needs to be tailored to the individual, that is, to learning styles, motivation (willingness and desire to change), and the ability of the offender (Andrews & Dowden, 2006) and based on their current lifestyle of behaviors.

### Implications for Future Research

The conceptual model developed needs to be tested across other groups of men who have used IPV to assess the model’s reliability, validity, and generalizability. This could be done by either using a deductive qualitative methodology where an a priori template of coding is used (Ryan & Bernard, 2003) or by the development of a questionnaire. It could also be extended to look at relationships where bilateral violence is a feature or where the violence is solely perpetrated by the female in the relationship. In addition, the conceptual model needs to be extended over time as desistance was only measured up to a year. Although this is clinically meaningful (Feld & Straus, 1989), it is not clear what stage of desistance this relates to and arguably does not represent secondary desistance but reflects a transitional phase, which is a necessary precursor of long-term desistance. Research therefore needs to be extended to include the longer term (i.e., over several years and perhaps decades) to understand how the mechanisms that underpin long-term desistance may differ from those identified in the model. Ideally desistance research should be conducted in community samples pre-and post-arrest but prior to the start of treatment and longitudinally over time. Researchers could then identify whether there are identifiable group differences between persisters and desisters prior to desistance being achieved, or whether these groups are a conceptual artifact of treatment and the desistance process. Using longitudinal data, researchers can examine historical, static, and dynamic factors that differ both initially between the groups and/or over time as this will aid our understanding of the process of desistance and thereby provide an insight to the potential targets for treatment.

### Limitations

Although this study provides a unique insight into the process of desistance from IPV which has to date been undertheorized, the findings need to be interpreted within the context of the study limitations. Group classification of the men was based on self-report using the Conflict Tactics Scale (CTS2; Straus et al., 1996), which can be problematic (Cook, 2002). There is no guarantee that the desisters had been violence free for a year. However, file notes were accessed for the desisters, which would have alerted the researchers to any police call-outs. Due to the proxy nature of police contact data as a measure of IPV (Falshaw, Bates, Patel, Corbett, & Friendship, 2003), it remains possible that although no-call out had been recorded for any of the desisters, physical violence may have occurred during this time frame. In addition, desistance was defined as an absence of physical violence by male perpetrators. It is acknowledged that IPV comprises a range of non-violent and coercive behaviors (Bowen, 2011), and that there is increasing awareness of the dyadic nature of IPV and specifically women as perpetrators (Johnson, 2006). Consequently, the present study potentially provides a limited picture of desistance from IPV, although findings are consonant with those reported previously in the literature (Gondolf & Hanneken, 1987; Scott & Wolfe, 2000).

It is also possible that response bias may have influenced the men’s reporting during the interviews. They may have been influenced to respond more positively about the role of treatment in light of the fact that the majority of interviews took place where the men were also attending treatment. It was observed that some of the men used language that is commonly used in treatment (e.g., the desisters talked about no longer being in denial and not using minimization as a technique). It cannot be guaranteed that what was being said was actually something that the men genuinely believed and had acknowledged and were putting into action, as they may have purely been recounting what they felt they should be saying based on their time in treatment. Likewise, some of the individuals interviewed may indeed be actually using these techniques, for example, denying and minimizing their use of violence, and so may have been distorting the version of events they were recounting. However, the parallel findings between these and those reported in previous studies would suggest that there is some validity to these findings across IPV perpetrator populations in different countries.

Although participants were recruited from different groups to give multiple perspectives, the sample was predominantly White British, which means that the experiences of other ethnicities were not included in the model. It has been suggested that cultural and ethnic differences affect how the process of desistance is experienced (Calverley, 2012), and so this needs to be considered in future research studies.

Finally, in qualitative research, it must be ensured as far as possible that the findings are the result of the experiences and ideas of the participants rather than those of the researcher (Ritchie & Lewis, 2003). In the current research, potential bias exists in that the researcher has previous research experience and knowledge of the topic area. It is possible that this may have influenced the way the data were collected and analyzed. However, to mitigate these potential biases, and in accordance with the guidelines proposed by Shenton (2004) to reduce bias and promote the credibility and confirmability of the research, a number of practices were used. First, the methodology and procedures used were derived from those successfully used in previous projects. Second, a colleague unfamiliar with the topic area was asked to verify that the questions were objective and consistent with the research objective. Third, detailed memos and extensive records were kept throughout each stage of the analytical process to provide evidence that the findings were data orientated and to demonstrate transparency regarding the development of the themes and resulting model. Fourth, systematic checks were also undertaken to ensure that the findings presented were clearly supported by evidence from the data, thereby accurately representing the experiences of the participants. Finally, two independent researchers, not involved in the research, were asked to examine and verify the analysis undertaken and the conclusions drawn.
